# The Place of Post-Traumatic Amnesia in the Assessment of Blunt Head Trauma: The Epistemic, Professional and Material Factors Shaping British Neurology, circa 1920–40

**DOI:** 10.1017/mdh.2018.42

**Published:** 2018-10

**Authors:** Ryan Ross

**Affiliations:** School of History, ArtsTwo, Mile End Road, Queen Mary, University of London, London E1 4NS, UK

**Keywords:** Head trauma, Post-concussional syndrome, Post traumatic amnesia, Neurology, Diagnosis, Second World War

## Abstract

The increase in road traffic accidents in twentieth-century Britain brought with it a rise in the number of patients admitted to hospital with blunt, non-penetrating head injuries. Patients who had suffered mild to moderate trauma typically complained of a variety of problems, including headaches, dizziness and giddiness. For the neurologists tasked with diagnosing and treating these patients, such symptoms proved difficult to assess and liable to obscure the clinical picture. This article focuses on why neurologists turned to time as a diagnostic-tool in helping to resolve these issues, specifically the measurement of post-traumatic amnesia (PTA). This article argues that PTA became so central to neurological diagnosis owing to a set of epistemic, professional and material factors in the decades prior to the Second World War. It concludes with a call for deeper appreciation of the range of issues that contribute to the shaping of medical theories of head trauma.

## 1 Introduction

From the interwar period onwards, members of the British medical profession connected a gradual rise in road traffic accidents with an increase in the number of patients being admitted to hospital with blunt, non-penetrating injuries to the head. The more serious traumata would result in death, coma or permanent disability, but physicians were also called upon to treat symptoms that developed from less severe injuries – post-traumatic sequelae that included headaches, dizziness or mild forms of amnesia. It was these symptoms that doctors found the most difficult to assess, for concussive sequelae bore no constant relationship to the severity of the head trauma. Furthermore, medics complained that a variety of psychological issues could enter the clinical picture to complicate diagnosis – a neurosis might develop if the impact of head injury sounded worse than it actually was or an anxiety disorder might emerge if the patient became overly-concerned with his future earnings or role as breadwinner.^1^ Indeed, it was even claimed that otherwise innocuous remarks – say, about how lucky the concussed patient was to have survived – might encourage the development of a complex.^2^ Things were only made worse if the patient brought an insurance claim or legal action – physicians believed that this might cause the deliberate or unwitting stimulation or exaggeration of symptoms, for, as one neurologist later commented in 1946, ‘The cumbersome machinery [of compensation] itself involves endless delays during which the [patient’s symptoms] become transformed into a “condition neurosis” in the sustained effort required in a fight for compensation’.^3^

These myriad concerns were regularly articulated in debates over head trauma and the so-called ‘post-concussional syndrome’ by the three physicians who are the focus of this article. For neurosurgeon Hugh Cairns, writing with neurologist Russell Brain in 1928, the archetypal patient (an injured workman, in this case) was impressed upon by a variety of individuals following head trauma, with detrimental psychiatric consequences. This included the doctor who informed the patient of his ‘narrow escape from a fractured skull’; the friends who identified the cause of his accident as another worker’s carelessness; and the employer who was unable to offer any light work while the patient regained his full strength. Anxiety neurosis was the outcome.^4^ Writing in 1941, neuropsychiatrist Charles Symonds expressed similar concerns about the patient’s recovery from mild to moderate head trauma. Echoing wartime concerns over military capacity, Symonds commented on the circumstances that may stymie a patient’s recovery, including ‘separation from friends, loss of promotion, [or] anxiety as to future capacity for service or civilian employment’. Indeed, as Symonds also acknowledged, insurance-claims and the medico-legal process could create all manner of difficulties for the diagnostician.^5^ For neurologist Ritchie Russell, the potential for cupidity was especially problematic, as it introduced a degree of unconscious motivation into the clinical picture.^6^ In fact, Russell found the issue of compensation so troublesome that he excluded patients with pending claims from his research on head trauma.^7^ It was better to focus on patients who had no pending claim, he asserted, for here the physician could limit the range of psychological factors at work in the sequelae of head injury.^8^

Cairns, Symonds and Russell were the most active British contributors to debates over the diagnosis and treatment of head trauma in the decades prior to the Second World War. Indeed, by 1940, the three of them would become involved in a pioneering neurosurgical hospital, the Oxford Military Hospital for Head Injuries.^9^ With recourse to published and archival sources, this article focuses on how Cairns, Symonds and Russell attempted to make more manageable the assessment of blunt head trauma in the context of the diagnostic problems identified above. In particular, I focus on how they made time central to neurological diagnosis by the beginning of the Second World War.^10^ My interest is in why the measurement of post-traumatic amnesia (or PTA) became so popular within British neurology in this period. PTA referred to a stage in the patient’s recovery in which he was conscious and aware of his surroundings but still confused as to where he was; the point at which this confusion ended, when studied in conjunction with the length of time that had elapsed from the accident, served as a proxy for assessing the severity of concussion (where the longer the duration of PTA, the greater the injury).^11^ By the 1940s, the measurement of PTA had crystallised as the most robust method for measuring the severity of head trauma, which in turn shaped the nature of the early research undertaken at the Oxford Military Hospital.^12^ My argument is that the importance of PTA stemmed from a combination of epistemic, professional and material factors.

This article begins by outlining a shift in medical thinking that occurred in the 1920s and which was associated by contemporaries with the surgeon Wilfred Trotter. I argue that concussion – the name given to transient loss of consciousness that left no long-term physiological damage – was reconceptualised by Trotter, leading to two broader developments: greater speculation that concussive sequelae were not psychologically but organically caused, and a new-found belief that recovery from concussion followed a specific model. The first task of this article is to show how Trotter gained such influence and how his model of recovery imbued a temporal dimension to neurological assessment of concussion.

Building on this, this article then shows how this temporal dimension to neurological assessment intersected with PTA and with the professional ambitions of Cairns, Symonds and Russell, for, by the late 1930s, neurologists and neurological surgeons argued that they were better placed than other medics to assess head trauma.^13^ Yet, the efforts by neurologists to examine concussed patients were often frustrated both by medical norms and by the difficulty of assessing the patient’s ‘subjective’ symptoms. Neurologists therefore turned to the use of PTA to overcome their difficulties and achieve an ‘objective’ assessment of blunt head trauma (it was, according to Cairns, the ‘best yardstick at present available’^14^). The second job of this article is to show how PTA became adopted by neurologists and neurosurgeons in the interwar years so as to facilitate their study of concussion.

The third aspect of this article focuses on the Oxford Military Hospital for Head Injuries. I identify the research undertaken there by Cairns, Symonds and Russell as pivotal in entrenching PTA within neurological thinking. It achieved this through the accumulation, and indexing, of patient-records for more than 1000 of the patients that it treated. For the purposes of this article, I want to draw attention to the two ways in which these records bolstered the neurological focus on PTA: through their explicit use in research publications by Cairns, Symonds and Russell; and through their composition, which privileged quantifiable neurological data like PTA. The third task of this article is show how the regime of data-gathering at Oxford was both product and producer of the temporal dimension to head trauma.

Through its focus on the epistemic, professional and material factors that made PTA central to neurological assessment, this article seeks to better develop the historical study of head trauma, which has thus far garnered only modest interest from historians.^15^ Those few studies to consider the subject have adopted a broad approach to stress continuity in the study of blunt head trauma, such that debates about concussion during the 1930s and 1940s echoed centuries-old tensions as to whether traumatic sequelae were organically or psychologically caused.^16^ The content of this article does not demur from this reading, offering a snapshot of a medical debate that had temporarily swung in favour of the organic theory,^17^ but the ambition of this article is to push beyond broad overviews of how medical thinking oscillated between organic and psychological theories of blunt head trauma over the past two centuries. Rather, by building upon recent studies of materiality and the neurosciences, I use this article to agitate for a richer, more fine-grained appreciation of how the development of medical theories of head trauma was imbricated within the particular circumstances of neurology and neurosurgery.^18^

## 2 The Epistemic Context: Wilfred Trotter’s Conceptualisation of Head Trauma

Trotter is today best known for his 1919 monograph on fear and group psychology, *Instincts of the Herd in Peace and War*.^19^ But his writings on concussive sequelae sparked far greater interest among his peers.^20^ In a series of publications and lectures after the First World War, Trotter began to speculate that concussion – the name given to transient loss of consciousness that left no long-term physiological damage – may cause longer-lasting injury than previously thought.^21^ Even minor degrees of head injury, he suggested, disrupted the circulatory system of the brain, contracting smaller blood vessels temporarily but in such a way as to produce physiological damage. As a consequence, Trotter argued, concussive sequelae like headaches were not always neurotic, as many within the medical profession believed, but could instead be related to unobservable organic damage to the brain.

Trotter’s first writings on the subject of concussive sequelae were initially published in 1914 as a lengthy chapter in a leading surgical textbook, Choyce’s *System of Surgery*.^22^ Various lectures and talks to learned societies then followed, many of which were later reproduced in the *British Medical Journal* or *The Lancet*. *Systems of Surgery* was republished in 1923, and then again with a third edition in 1932.^23^ Despite the interval between these chapters, Trotter’s theory of concussion remained largely unchanged, and his chapter in the 1932 edition of *Systems of Surgery* was well-cited in the decade following its publication. In all of his writings on head trauma in this period, Trotter adopted a deductive approach: he did not undertake any original anatomical research of the brain to derive his theory, but instead extrapolated both from published studies of severe head trauma and from physiological research into cerebral circulation. On this, of course, Trotter had form – his *Instincts of the Herd in Peace and War* was originally written in a spirit of academic enquiry, not as the result of anthropological field-work.^24^

Trotter’s theory of concussion began with a rejection of neurosis as cause of post-concussional symptoms.^25^ His argument was premised on a belief that a concussed patient needed to possess a near-complete memory of their original trauma in order to subsequently develop a state of fear or anxiety. This was often denied to the concussed, however, for they frequently had amnesia for the events leading up to their head injury. Without a reliable memory of their trauma, Trotter reasoned, the concussed patient could not develop neurotic symptoms.^26^

This led Trotter to reject neurosis as cause of the post-concussional syndrome and to advance an organic theory instead. It pivoted on three points. Focusing on post-traumatic headache, which he regarded as the ‘most characteristic’ of the concussive sequelae (and able, as such, to stand for other symptoms like giddiness), Trotter hypothesised that intracranial pressure was its primary cause. Whilst conceding that much remained unknown about minor head injuries, intracranial pressure, he noted, if distributed inequitably, altered the tension between the dural septa, with headache the consequence.^27^ Indeed, he identified disturbance of brain-circulation as central to the development of cerebral symptoms, as had already been shown by notable physiologists and surgeons like Horsley, Hill, Kocher and Cushing.^28^ Because of this, Trotter pointed to the brain’s location within the skull as a source of distress, for when an organ needs to release a build-up of blood, he observed, it swells in such a way as to carry away any excess and thus to restore circulation to normal functioning. However, the brain – uniquely, Trotter argued – was often unable to do this as fully as it needed to, as the space between it and the skull was restricted and could not be easily enlarged. As a consequence, Trotter claimed, the brain could be unable to reabsorb excess fluid, in some cases for several years following contusion, and which could lead, in even minor injuries, to the development of headaches and associated symptoms.^29^ This build-up of fluid, Trotter concluded, was reversible and would resolve itself naturally in most cases, but for this required the passage of time.

## 3 The Popularity of Trotter

Trotter’s peers took no issue with the model of recovery he proposed. What they did not accept was the circulatory (or vascular) theory he advanced as the cause of post-concussive sequelae. For example, neurosurgeon Geoffrey Jefferson faulted it on the grounds that Trotter could not explain what caused long periods of unconsciousness, beyond a general bruising of the brain (Jefferson’s view was that concussive sequelae were caused by brain-stem lesions).^30^ Elsewhere, Symonds criticised Trotter’s vascular theory because, he argued, concussive sequelae were not comparable with other disorders caused by intracranial pressure – they were not, he noted, characterised by ‘irritability, restlessness, and confusional excitement’ as found in concussed patients. Symonds further observed that his histological studies had demonstrated no clear correlation between intracranial pressure and the severity of the symptoms.^31^ Russell criticised Trotter on this point too, arguing that the vascular theory did not sufficiently explain discrepancies in the duration of unconsciousness between certain cases. According to this theory, mild injuries should recovery rapidly, yet Russell had encountered many cases where that was not the case. Like Symonds, he instead advocated for a theory of head trauma that emphasised neuronal loss as cause of concussive sequelae.^32^

Despite these criticisms of Trotter’s vascular theory of concussion, he nevertheless held considerable respect among contemporary medics for proposing an organic basis to post-concussive symptoms. He was celebrated for ‘rescuing’ concussed patients who had previously been stigmatised as neurotic. Trotter’s model of recovery was also praised, even by those who demurred from his vascular theory of concussion, such as Symonds, Jefferson and Russell.^33^ Indeed, Trotter received considerable praise from Symonds, who later positioned the surgeon as the starting-point in the gradual (and progressive) evolution in theories of concussion.^34^ Yet there is an important point to be made about exactly when Trotter became popular, for his earliest writings in the 1920s were but modestly referenced by anyone other than Symonds. It was not until the mid-1930s, and after, that Trotter’s theory of concussion became implicated in broader medical debates about blunt head trauma, accidents and the role of compensation in prolonging traumatic sequelae. The reason for this is to be found in the epistemic context of the period. This framed concussion in a particular way, based on a particular set of priorities, and is best explored with reference to the complaints made of one of the labels applied to concussive sequelae, that of traumatic neurasthenia.

Neurasthenia was a disorder that pre-dated the twentieth century, where its defining symptom was fatigue.^35^ By the interwar period, however, neurasthenia was reframed in psychological terms, and regarded essentially as an anxiety neurosis, a state of intense fear of an unspecified future danger and which could follow any physical injury.^36^ Given the linking of concussion with road traffic accidents and systems of compensation, neurasthenia, when it was applied to head trauma, implied that the sufferer’s concussive sequelae were hysterical or outright fraudulent. Because of this, some doctors fretted that concussed patients with otherwise genuine physical injuries could have their symptoms dismissed as cupidity or be cast onto the ‘dumping ground’ of neurasthenia.^37^ Hence the concern, iterated throughout the 1930s, that neurasthenia was a misleading, and potentially disastrous, medical diagnosis.^38^ Indeed, writing in 1931, psychotherapist Millais Culpin cautioned other doctors against labelling atypical concussive sequelae as neurasthenic or related to financial remuneration, claiming that he had observed post-concussive anxiety in cases where compensation was not an issue or had already been settled.^39^ He went so far as to suggest that compensation was only clung to by the concussive patient as a coping-mechanism in light of their fears for the future and of the disdain they were held in by many members of the medical profession.^40^

In Culpin’s model of concussive sequelae, psychological factors were superimposed onto the organic trauma, magnifying or extending sequelae beyond what was regarded as their natural point of termination – in the same way that a neurosis could develop from an injury to any other part of the body because of fears of permanent disablement. This aetiological model was popular and could be easily applied to injuries with a clear physiological basis, including concussion once Trotter’s organic theory was accepted. Indeed, as psychiatrist Edward Mapother argued, to suggest that neurasthenia and the post-concussional syndrome were separate disorders would mean having to explain how two different pathologies produced identical symptoms (a task that Mapother regarded as ‘very doubtful’).^41^

The attraction of using this psychogenic model of concussive sequelae was that it could bind together strands of medical thought that were elsewhere in tension. For example, it allowed doctors to side-step long-running debates about organic-vs-psychological causes of the post-concussional syndrome: a head injury was always both, they now argued.^42^ More significantly, the psychogenic aetiology allowed doctors to duck contentious arguments about the actual damage concussion caused to cerebral function, for, although thinking began to consolidate around the organic theory of concussive sequelae by the 1940s, doctors were no closer in agreeing what the precise mechanism was by which head trauma disrupted cerebral function.^43^ Trotter’s explanation focused on circulatory pressures within the brain, but it was argued that it left questions unanswered.^44^ Symonds proposed an alternative theory of concussion based on neuronal damage, which was met with modest enthusiasm.^45^ It need not have mattered, however. Trotter’s organic theory of post-concussional syndrome did not need to explain precisely how concussion affected brain function. A belief that *something* had occurred was sufficient.

Indeed, the real advantage of Trotter’s theory was that it allowed doctors to attribute all manner of concussive sequelae to psychological factors.^46^ This is evident in the writings of Symonds, Trotter’s most vigorous champion during the interwar period.^47^ For Symonds, the brilliance of Trotter’s organic theory of post-concussional syndrome was that it accommodated the place of fear in its genesis. Writing in the 1930s, and reflecting a broader medical interest in the subject, Symonds began to underline the role of fear and anxiety in the aetiology of post-concussive sequelae, highlighting how psychological factors could be overlaid onto even mild head trauma.^48^ The attraction to Trotter’s organic theory of concussion was that it allowed for these issues to be amply accommodated within the medical diagnosis.

## 4 ‘Stage Theory’ and the Importance of PTA to Neurological Assessment of Blunt Head Trauma

The effect of Trotter’s reconceptualisation of concussion was to imbue a temporal dimension to recovery from blunt head trauma, as was evident in the growing use of so-called ‘stage theory’ from the 1930s. Building upon Trotter’s work, this held that, irrespective of the severity of most injuries (and barring death or deep coma), all concussed patients recovered in the same way, by passing through a series of stages that varied only by the rate of their progression – they moved, as Cairns wrote in 1942, from a state of unconsciousness, to one of confusion, to another characterised by intermittent headaches, before culminating in ‘restor[ation] to normal or near-normal’.^49^ Because of the universality of the stages, a measurement of the speed at which the patient progressed through them emerged by the 1930s as a proxy for assessing the severity of the original head trauma; and knowledge of the original trauma prevented the neurologist from being misled or diverted by any psychological disorders or neuroses that the patient had developed post-accident.

The problem for the neurologist, however, was that he was rarely in a position to measure the patient’s progress through every stage. Writing in 1942, Symonds conceded that physicians in general faced tremendous challenges in treating concussed patients, as it proved difficult to assess the severity of an injury when ignorant of the circumstances surrounding the accident – challenges magnified when the patient was unconscious or unable to recollect anything of what had happened to them.^50^ Neurologists had even harder luck: typically, they were the last to see concussed patients after surgeons and casualty-officers, and often had to wait several days – sometimes weeks during wartime – before the patient was transferred to them. By this point, the concussed patient had been assessed by a variety of medics who held various degrees of specialism, had documented head trauma with their own nomenclature, had their own set of priorities (the most immediate for surgeons being to resuscitate the comatose patient) and did not always collect information of later use to neurologists (ie. how the patient progressed through the early stages of recovery).^51^ It was a record of the early stages that proved particularly frustrating for neurologists like Symonds and Russell, as they acknowledged that most neurological and psychometric tests were of little use too early in the patient’s recovery: more basic information was required as to the occurrence of symptoms, for instance, or the length of unconsciousness. Indeed, given the lack of consensus on precisely how concussion caused physiological damage, it meant that X-rays and other physical tests (eg. measurement of intracranial pressure or of cerebrospinal fluid) were not regarded as essential.^52^

It is little surprise, therefore, that in the 1930s neurologists were calling for the acquisition of greater information on concussed patients, of the sort that the average surgeon would not normally be concerned with – eg. the time and place of the accident, or the mental state of the patient after admission (plus his response to stimuli and commands). As Symonds explained, the neurologist required a speedy means of charting the sequencing of the patient’s early recovery that simply contained ‘the main facts’, similar to a pulse or respiration chart.^53^ Furthermore, neurologists argued that observational data was required on the patient on the days and weeks following their recovery to gauge progress, in combination with other tests such as X-rays.^54^ There was thus a noticeable push by neurologists, beginning in the 1930s, to castigate general surgeons for not adequately assessing and documenting concussed patients.^55^ There was an attempt, in so doing, for neurologists to enfold concussed patients into their own professional remit.^56^ Surgical intervention was rarely needed, neurologists argued, and only then in the first twenty-four hours of recovery.^57^ After this point, they averred, the patient was best served by a specialist rather than a general surgeon.^58^ Indeed, as Cairns observed in 1940, documentation was central to the treatment of the concussed patient; neurologists were identified as the best people to compile it.^59^

It was within this context that Symonds and Russell began to advocate for the importance of the measurement of PTA in the assessment of head trauma.^60^ It was the latter who, in 1932, first wrote of its usefulness, borrowing explicitly from Trotter’s stage theory to identify PTA as an effective gauge of the severity of an injury to the head.^61^ That the measurement of PTA did not require knowledge of the accident nor immediate access to the comatose patient made it particularly apposite for neurological assessment. Indeed, by the start of the Second World War, neurologists began to make a virtue out of their lack of immediate access to the patient – writing in 1940, Symonds noted that concussion could only be measured retrospectively, for a length of time was necessary to allow the concussive sequelae to develop.^62^ Furthermore, in the writings of Symonds and Russell, PTA began to be associated with a more scientific approach to the assessment of blunt head trauma, as if it could act as an empirical buttress against the subjectiveness of the post-concussional syndrome.^63^ After all, measuring the length of the patient’s unconsciousness, through minutes and hours, was easy to quantify and standardise, and required no more expensive technology than a wrist-watch.

This is not to suggest that PTA did not have its limitations. Indeed, there was some initial disagreement as to how PTA should be measured and used. As Russell conceded, it could be hard to determine when a patient had moved from a stage of confusion to one of lucidity, for some confused patients could nevertheless speak intelligibly.^64^ Moreover, the use of PTA also brought with it a number of more specific warnings by Russell and Symonds: it could not address complications like haemorrhage or local injuries (ie. to the skull) and must not be used in isolation from other tests and assessments.^65^

Despite these limitations, by the Second World War PTA had become central to the neurological assessment of blunt head trauma as advocated by Symonds and Russell. There is some indication in the contemporary medical literature as to why this was, and which explains why neurologists wanted to achieve the standardisation of the assessment of head injury, because neurologists wrote in the 1930s of the real difficulty they faced in their assessment of concussed patients. This was the patients themselves or, more accurately, their emotional baggage.^66^ As I have already shown, many physicians acknowledged the role of psychological factors in the development of concussional sequelae. The challenge for neurologists came from seeking to differentiate between psychological overlay and organic damage. Moreover, with so many concussional symptoms labelled ‘subjective’ (eg. headaches, dizziness), the neurologist was reliant on the patient in assessing the extent of injury – an unreliable source if that patient also hinted at psychological issues.^67^ The financial wish for compensation, in particular, was identified as especially problematic, introducing a degree of unconscious motivation into the range of factors that physicians had to take into account.^68^ Moreover, neurologists expressed frustration with the subjectiveness of psychiatric nomenclature: the difficulties they faced in collecting information on patients was confounded by what was identified as a lack of clearly-defined terminology for describing the sequelae of concussion. The neurologist’s task of trying to make sense of it all was a source of irritation, though one which they were as much a cause of as any other medical specialism: following the writings of Trotter, many neurologists began to question what the terms ‘concussion’ and ‘post-concussional’ actually meant (it was noted that the former implied a transience that may be unjust, particularly in cases involving long-term concussive sequelae).^69^ Hence the reason why many doctors proposed new definitions of concussion, or new terms altogether (eg. ‘cerebral contusion’, ‘traumatic psychasthenia’).^70^ It was also for this reason, according to Symonds, that the Brain Injuries Committee of the Medical Research Council (MRC; of which he and Cairns were members) produced a list of medical terms at the start of the Second World War, to better standardise the language that neurologists and other physicians used in writing about head trauma.^71^

## 5 The Data-Collection Regime of the Oxford Military Hospital for Head Injuries

It was within this context that the measurement of PTA became central to neurological assessment of head trauma. Yet, the professional ambitions of neurologists also led them to create a data-collection regime of the Oxford Military Hospital which privileged what were perceived as ‘objective’ and standardised elements of neurological assessment.^72^ The hospital began admitting patients from February 1940 onwards, with Cairns, Symonds and Russell on the medical staff from the outset.^73^ From early in the hospital’s development, many medics wrote widely of the opportunities that such a neurosurgical centre created.^74^ Of particular importance in this period were the activities of the Brain Injuries Committee of the MRC, which first met in March of 1940 and which comprised eminent neurologists and neurosurgeons, including Cairns and Symonds.^75^ It had the ability to review grant-applications and to make funding recommendations to the MRC. In anticipation of the Oxford Military Hospital opening, the committee’s members suggested a number of potential ‘lines of enquiry’ for neurologists based at or close to Oxford to pursue – for instance, an electroencephalogram (EEG) analysis of patients who developed traumatic epilepsy, or a study of the efficacy of various antiseptics on brain tissue.^76^ Oxford’s potential was not lost on Symonds, who, along with other committee members, sought to underscore the boost to medical research that the Military Hospital could create if appropriate administrative steps were taken.

Symonds proposed that the Military Hospital should mandate physicians to take detailed notes on patients, and then have these notes ‘filed, indexed and kept at the hospital, as a basis for research along whatever lines appear most profitable, as the material accumulates’.^77^ He successfully acquired funding from the MRC so as to employ one full-time secretary and purchase the necessary Dictaphones, typewriters and filing-cabinets to properly document and organise the patient-records.^78^ Furthermore, following discussions that originally began in 1940, and which included the Ministry of Pensions, Cairns and Symonds planned long-term, follow-up studies with patients, with a view to ascertaining the presence or abeyance of symptoms at six months from discharge. This initially caused some anxiety for Symonds and Cairns, for how were they to create an index to be used at some stage in the future for research that could develop in any direction? As Cairns wrote to Geoffrey Jefferson in July 1940, ‘…it is quite a difficult business to formulate questions for a statistical enquiry that will be of value 10 years hence…’.^79^ Robust data was therefore needed, and, in planning the data-collection regime of the hospital, Cairns and Symonds set out to future-proof the system. The hospital was thus not only to treat patients, but to accumulate their records for current and future researchers.

To achieve this, Symonds proposed an extensive system of record-keeping for physicians and medical officers at the Military Hospital, which was to be kept separate from army or pension records.^80^ Under this plan, doctors were to record the patient’s history and the medical examination in the same way in every case, with Symonds supplying physicians with extensive criteria for documentation. Thus, the written record of the patient’s history was to include details of their memory (last recollection before the injury, first memory after), their present complaints, their family history (of epilepsy or migraines, and including ‘mental or nervous instability’ in close relatives) and the patient’s personal history (eg. school performance, employment history, hobbies, significant other(s) and any past medical illnesses).^81^ The physical examination of the patient was to be similarly extensive, incorporating a neurological and general assessment of the patient, plus an evaluation of their mental state (their behaviour, mood, preoccupations and of their ‘intellectual functions’ like memory and calculation).^82^

This accumulation and organisation of patient-records at the Oxford Military Hospital was to have deeper ramifications for the use of ‘objective’ criteria in neurological research. As I have explained, Symonds sought to acquire extensive information on every patient, incorporating a record of their history and their neurological examination, and he provided written instructions to medics to secure this.^83^ Symonds’s wish to record this information in every patient admitted to the Military Hospital thus had the effect of standardising the process of record-keeping.^84^ This process of standardisation did not stem from mere fussiness on the part of Symonds, however. Rather, it was an essential function of neurological research.^85^ If a researcher wanted to quantify the relationship between certain criteria (say, between the site of a head injury and the severity of later symptoms), then they required complete data on the presence/absence of that criteria in a robust number of patients.^86^ For research to be undertaken reliably, the data collected had to comparable and clearly defined, and this meant standardising the process of record-keeping.^87^

In seeking to correlate patient-data, neurologists at Oxford therefore made use of a database (of sorts) – a paper database, that is, composed of punch-cards.^88^ These were compiled from a process involving several stages. The first stage was for the secretary to transcribe the handwritten patient-records. The typewritten record was then indexed by Symonds, who undertook a review of each patient-record; the indexes that he compiled were then passed to Russell, who, after a further check, transferred the data to punch-cards (or Paramount cards, as they were sometimes referred to). As Russell later wrote: ‘In order to facilitate analysis the main facts of the records [were] transferred to Paramount Cards. These particulars had previously been indexed in each case by Air Commodore Symonds, and were transferred by myself after a further check to the cards. This ensured a relatively uniform assessment of the main facts.’^89^

A punch-card was thus created for each of the first 1000 consecutive patients admitted to the Oxford Military Hospital, which was intended to give a researcher immediate access to the range of information contained in any one patient’s corresponding record.^90^ Along the four edges of each card was an array of perforations, each one corresponding to an aspect of the patient’s symptoms or to their pre- and post-traumatic state. Thus, there were perforations corresponding to the site of the head injury (one perforation each for qualities like ‘Frontal’, ‘Temporal’, ‘Brain Stem’, etc.); perforations for aspects of the patient’s mental examination (one each for symptoms like ‘Elation’, ‘Apathy’, ‘Hysteria’, etc.); perforations relating to family history (one each for ‘Mental’, ‘Epilepsy’, ‘Migraine’); and perforations corresponding to the length of post-traumatic amnesia (one for ‘Nil’, ‘<1 Hr’, ‘1–24 Hr’, ‘Delayed’, etc.).^91^

Altogether, each punch-card contained over 160 perforations; and if the patient possessed, say, a family history of migraine or suffered from a brain-stem injury, then the relevant perforation would be slightly larger (accordingly, the perforation for ‘Migraine’ and ‘Brain Stem’). Were a researcher to arrange these cards so that their perforations aligned, patients in possession of particular symptoms could be easily identified by looking for the wider perforation that corresponded to the symptom under review (the researcher could trace the widened perforations along a stack of cards with ease, as several widened perforations together leave a striation).^92^ Thus, when arranged together, the punch-cards guided the researcher to the relevant patient-records, in effect mediating the researcher’s access to the original record: no longer did the researcher need to peruse all 1000 patient-records to identify, say, all those patients with *contre-coup* injuries; instead, the researcher simply examined the punch-cards to learn the names or patient-numbers of the relevant records.^93^ With the names/numbers of patient-records in hand, the researcher could simply collect their record from the appropriate filing-cabinet. This system of punch-cards allowed researchers at Oxford both to undertake large statistical studies of patients who had multiple medical problems (post-concussive migraines and brain-stem injuries, for example) but also, it was hoped, to identify a ‘new clinical syndrome’: ‘A case, for example, may appear in which there is a striking association between delayed traumatic amnesia and slow pulse rate. The observer [researcher] may by means of the index [punch-cards] easily obtain and review the notes of all cases in which this particular combination has occurred.’^94^ The punch-card was thus central to research conducted by the Oxford neurologists. It was, in effect, the gatekeeper for the patient-records.

## 6 Publications and PTA

The data-collection regime at the Oxford Military Hospital would benefit researchers for decades after the War, and in a way that stretches beyond the scope of this article.^95^ Yet, it is worthwhile to briefly draw attention to the effect of the data-collection regime at Oxford on the place of PTA within neurological thinking in the early years of the Second World War. Indeed, as much as the data-collection undertaken at the hospital reflected the new place of PTA within neurological thinking, it also helped to reinforce it.

In publications during the 1940s, Cairns, Symonds and Russell continued to underline the usefulness of PTA for neurological assessment. Just as with Russell’s earlier publications on the usefulness of PTA, the Oxford neurologists sought to classify patients based on the severity of their head trauma. Both sets of publications – Russell’s in the early 1930s, and those by Cairns, Symonds and Russell in the 1940s – used PTA identically. What changed was the context in which both sets of publications were produced, for the research from the 1940s was primarily concerned with the usefulness of PTA in assessing prognosis (or the earliest opportunity at which the patient could be returned to front-line duty).^96^ It is in these publications from the 1940s that we can observe the impact of the military context on neurological research – the needs of war heightened the assessment of prognosis and rehabilitation of the concussed, and PTA was to be used to judge this. Indeed, the need for rehabilitation became more prominent in medical thinking as the war progressed, and particularly following the publication of the Beveridge Report in 1942 and the Inter-Departmental Committee on Rehabilitation in 1943. Indeed, from 1940 onwards, Symonds and Cairns pressed the Ministry of Pensions for the construction of neurosurgical and rehabilitation centres for use post-war^97^ This wartime context is evident in other ways, for one of the puzzles regarding the Oxford research on PTA is why it was ever published in the first place. Russell’s work from the early 1930s had already demonstrated – as both Symonds and Cairns conceded – that PTA was a valuable means of gauging the severity of an injury. No was any criticism levelled against Russell by either Symonds or Cairns in the 1940s, nor did Russell row back on his earlier argument.

The military context explains why neurologists nevertheless sought to demonstrate the value of PTA all over again. As Cairns wrote, blunt head trauma had become more prevalent during the war, and was subject, he argued, to treatment by greater numbers of physicians, none of whom had undergone the same degree of training or had kept abreast of recent medical developments.^98^ There was a need, therefore, for a greater education of medical audiences, to rectify what Symonds and Russell identified as ‘uncertainty among surgeons and physicians regarding the prognosis in these cases’.^99^ It was for this reason, I suggest, that Russell republished his earlier work on PTA in the *British Medical Journal* in 1942, which he prefaced as being of ‘practical use to help those who treat these cases [of head injury] and to those who as yet have made no special study of the subject’.^100^ There may also have been further requests for knowledge directed at the Oxford neurologists too, as, according to Symonds and Russell, the encouragement of others within the medical establishment lay behind their decision to publish some preliminary comments on the treatment of concussion in 1943.^101^

Yet, it is also worth considering the punch-cards used by Cairns, Symonds and Russell and their impact *vis-a-vis* neurological assessment. The system of indexing utilised at Oxford imposed a structure to patient-records, circumscribing how they were subsequently accessed by other researchers.^102^ For a symptom to be later studied, it had to be recorded on the punch-card. As patient-records at Oxford were compiled in anticipation of their eventual indexing, a particular shape was imposed upon them. For example, symptoms had to be succinctly expressed so as to fit on a punch-card. They also had to be standardisable, able to be deduced from the patient and recorded in every case (the same template was used for every card). Finally, symptoms had to be clearly proven – a patient either developed meningitis or not; they either had a penetrating head injury or they did not; they either possessed a pre-existing history of epilepsy or they did not. There was no place for nuance or ambiguity on the punch-card; and, in such a system, only two qualities were privileged – incidences and quantification. PTA, which was measured through quantification (eg. hours and days), was therefore afforded a high degree of stability. PTA thus became central to neurological assessment not only because of an epistemic shift and the professional ambitions of Cairns, Symonds and Russell, but also because it was easily represented and understood by the regime of data-collection introduced at the Oxford Military Hospital.

## 7 Conclusion

The centrality of PTA to the assessment of blunt head trauma is thus explained with reference to the epistemic, professional and material factors influencing British neurology in the decades prior to the Second World War. This article focused on Wilfred Trotter’s reconceptualisation of concussion in the 1920s, showing how his organic theory of head trauma gained considerable influence. Notwithstanding misgivings over his vascular hypothesis, Trotter was able to reconfigure how the medical profession viewed concussive sequelae, encouraging medics to understand head trauma as an organic process and not simply as a psychological one. Trotter’s theory also imbued a temporal dimension to medical thinking, encouraging the wider use of ‘stage theory’ to interpret recovery from concussion.

As this article demonstrated, the temporal dimension of neurological diagnosis then led to the greater use of PTA by neurologists and neurosurgeons like Cairns, Symonds and Russell. They complained in the 1930s about how frequently specialists were frustrated in their efforts to gain immediate access to concussed patients, and, in any event, found the patient difficult to diagnose owing to the psychological issues that followed the trauma. Within this context, PTA emerged as a useful tool for neurologists to use, requiring no immediate access to the patient and perceived as an objective buttress against the patient’s subjective symptoms. Finally, this article showed how the material objects used to research blunt head trauma by Cairns, Symonds and Russell both reflected the new place of PTA in medical diagnosis and also helped to consolidate it. I drew attention to the data-collection regime at the Oxford Military Hospital for Head Injuries where Cairns, Symonds and Russell were based during the Second World War, documenting how this privileged quantifiable neurological data. The result was the further entrenchment of PTA within neurological diagnosis.

The implications of this article on the extant historical literature are two-fold. On one level, it complements the growing research on the relationship between scientific knowledge and the professional and material context from which that knowledge is shaped. At the same time, this article offers a more pointed intervention in historical studies of head trauma. Granted, this article’s focus offers further evidence for what historians have identified as the centuries-old debate as to whether traumatic sequelae were organically or psychologically caused – a debate that was never fully resolved by the end of the twentieth century.^103^ Indeed, when read against the debates that would characterise postwar Britain neurology, it could be argued that the acceptance of Trotter’s organic theory of head trauma was only temporary.^104^ There is something to be said, perhaps, for studying the long-term development of medical thinking on blunt head injuries, of setting the scene or offering broad overviews. The content of this article could be accommodated within these broader studies.

Yet, through its narrower focus on Cairns, Symonds and Russell, this article has shown how shifts in medical theories of head trauma were imbricated with a wider range of issues affecting neurologists and neurosurgeons (in this case, professional and material factors). My point is that developments in medical thinking occurred not merely at the level of the epistemic, either through force of logic or the acceptance of one school-of-thought over another; rather, ideas gained influence because they reflected (and were reflective of) a larger range of issues. Broad histories of head trauma that focus on epistemic change alone risk overlooking the more specific interests that prompt (and then secure) developments in medical understanding. This article recognises that there may be a place for big narratives, but it also demonstrates the value of attuning to the more specific contexts in which developments in medical thinking occur.

## Footnotes

1.
W. Ritchie Russell, ‘Medical Aspects of Head Injury’, *British Medical Journal*, 2, 4269 (1942), 521; J.G. Danson, ‘On Injuries to the Brain and their Sequelæ’, *Proceedings of the Royal Society of Medicine*, 33, 2 (1939), 60; Charles Symonds, ‘The Assessment of Symptoms Following Head Injury’, *Guy’s Hospital Gazette*, 51 (1937), 461–8, reprinted in Charles Symonds, *Studies in Neurology* (London and New York: Oxford University Press, 1970), 122. Note that the accident-sufferer was always identified as male within the medical literature.10.1136/bmj.2.4269.521PMC216451420784518

2.

J.P. Martin , ‘Mental Symptoms Associated with Head Injury’, British Medical Journal, 2, 4012 (1937), 1062.10.1136/bmj.2.4012.1061PMC208777320781075

3.
James K. Slater, ‘Trauma and the Nervous System, With Particular Reference to Compensation and the Difficulties of Interpreting the Facts’, *Edinburgh Medical Journal*, 53, 11 (1946), 641. On medical debates over malingering, see Roger Cooter, ‘Malingering in modernity: psychological scripts and adversarial encounters during the First World War’, in Roger Cooter, Mark Harrison and Steve Sturdy (eds), *War, Medicine and Modernity* (Sutton: Stroud, 1998), 125–48. On the difficulties faced by physicians in deducing malingering in the nineteenth and twentieth centuries, see Edward M. Brown, ‘Between cowardice and insanity: shell shock and the legitimation of the neuroses in Great Britain’, in Everett Mendelsohn, Berritt Roe Smith and Peter Weingart (eds), *Science, Technology and the Military* (Dordrecht and Boston: Kluwer Academic Publishers, 1988), 323–5.; Karl Figlio, ‘How does illness mediate social relations? Workmen’s compensation and medico-legal practices, 1890–1940’, in Peter Wright and Andrew Treacher (eds), *The Problem of Medical Knowledge: Examining the Social Construction of Medicine* (Edinburgh: Edinburgh University Press, 1982), 174–224.PMC528341420282671

4.

W. Russell Brain and Hugh Cairns , ‘The Treatment of the Sequels of Head Injury’, The Lancet, 211, 5457 (1928), 668.

5.

C.P. Symonds , ‘Closed Head Injuries’, Journal of the Royal Medical Corps, 76 (1941), 327.

6.

C.P. Symonds , ‘Concussion and contusion of the brain and their sequelae’, in Brock Samuel (ed.), Injuries of the Brain and Spinal Cord (London: Baillière, Tindal & Cox, 1940), 101.

7.
For examples of Russell’s exclusion of compensation cases, see W. Ritchie Russell, ‘Cerebral Involvement in Head Injury: A Study Based on The Examination of Two Hundred Cases’, *Brain*, 55, 4 (1932), 584 and 592; W. Ritchie Russell, ‘The After-Effects of Head Injury’, *Transactions of the Medical-Chirgical Society of Edinburgh*, 48 (1933–34), 134–5; Russell, *op. cit.* (note 1), 521.

8.
See his comments in Russell, ‘Cerebral Involvement in Head Injury’, *ibid.*, 602.

9.
This was based at a repurposed St Hugh’s College. The historical literature on the hospital remains somewhat scanty, however, and usually only crops up in biographies of Cairns, eg. G.J. Fraenkel, *Hugh Cairns: First Nuffield Professor of Surgery, University of Oxford* (Oxford: Oxford University Press, 1991), esp. ch. 11; J. Tailor and A. Handa, ‘Hugh Cairns and the Origin of British Neurosurgery’, *British Journal of Neurosurgery*, 2, 1 (2007), 190–6; Peter H. Schurr, ‘The Evolution of Field Neurosurgery in the British Army’, *Journal of the Royal Society of Medicine*, 98, 9 (2005), 423–7; J. van Gijn, ‘Sir Charles Symonds (1890–1978)’, *Journal of Neurology*, 260, 4 (2013), 1191–2.

10.
On the growing role of time in medical diagnosis throughout the twentieth century, and the connection of this to patient-records, see David Armstrong, *A New History of Identity: A Sociology of Medical Knowledge* (Basingstoke: Palgrave Macmillan, 2002), 140 and *passim*.

11.
For an outline of it, see Cairns in [no author], ‘Discussion on Rehabilitation After Injuries to the Central Nervous System’, *Proceedings of the Royal Society of Medicine*, 34, 4 (1942), 299; Russell, ‘Cerebral Involvement in Head Injury’, *op. cit.* (note 7), 533. On the neurological belief that the patient’s outward signs revealed internal processes, see Stephen T. Casper, ‘The patient’s pitch: the neurologist, the tuning fork, and textbook knowledge’, in L. Stephen Jacyna and Stephen T. Casper (eds), *The Neurological Patient in History* (Rochester, New York: Rochester University Press, 2012), 21–43.

12.
Note my focus on the *early*research undertaken at the Oxford Military Hospital. Later research ranged over a variety of areas, including the treatment of gun-shot wounds to the head and the use of penicillin in neurosurgery. However, these remain outside the scope of this article, which is concerned with blunt head trauma only. For a broader overview of the hospital’s activities during the War, see Hugh Cairns, ‘Investigations on Head Injuries in Britain During the Second World War’, *Schweizerische Medizinische Wochenschrift*, 76, 37–8 (1945), 865–70.21000469

13.
For broader context on the place of neurologists in this period, see Stephen T. Casper, *The Neurologists: A History of a Medical Speciality in Modern Britain, c. 1789–2000* (Manchester: Manchester University Press, 2014), esp. ch. 3. For neurosurgeons and their relationship with neurologists in Britain, see Delia Gavrus, ‘Skill, Judgement and Conduct for the First Generation of Neurosurgeons, 1900–30’, *Medical History*, 59, 3 (2015), 361–78; Anna Kathryn Schoefert, ‘Neither Physicians Nor Surgeons: Whither Neuropathological Skill in Post-War England?’, *Medical History*, 59, 3 (2015), 404–20.10.1017/mdh.2015.27PMC459724126090736

14.
No author, *op. cit.* (note 11), 299.

15.
As argued in Ryan Ross, ‘Accidents and Their Medico-Legal Sequelae in Twentieth-Century Britain: Time, Trauma and Writing History’ (unpublished PhD thesis: University of London, 2017).

16.
Historians have charted the tensions between organic vs psychological theories of trauma throughout the nineteenth and twentieth centuries, and in debates over disorders like railway spine, shell-shock and the post-concussional syndrome. See Edgar Jones, Nicola T. Fear and Simon Wessely, ‘Shell Shock and Mild Traumatic Brain Injury: A Historical Review’, *The American Journal of Psychiatry*, 164, 11 (2007), 1641–5; Stephen T. Casper, ‘Concussion: A History of Science and Medicine, 1870–2005’, *Headache*, 58, 6 (2018), 795–810; Michael R. Trimble, *Post-Traumatic Neurosis: From Railway Spine to the Whiplash* (West Sussex: John Wiley & Sons, 1981), ch. 5; Tracey Loughran, *Shell-Shock and Medical Culture in First World War Britain* (Oxford: Oxford University Press, 2017). A similar focus has characterised histories of whiplash: eg. Robert Ferrari and Edward Shorter, ‘From Railway Spine to Whiplash: The Recycling of Nervous Irritation’, *Medical Science Monitor*, 9, 11 (2003), 27–37. On how the same set of issues have dogged the neurosciences more generally in the past century, see Michael Hagner and Cornelius Borck, ‘Mindful Practices: On The Neurosciences in the Twentieth Century’, *Science in Context*, 14, 4 (2001), 507–10.

17.
I call this support of the organic theory ‘temporary’ as an attempt was made to espouse the opposite, psychogenic theory of concussive sequelae a few decades later. See Ryan Ross, ‘Between Shell Shock and PTSD? “Accident Neurosis” and Its Sequelae in Post-War Britain’, *Social History of Medicine* (in press).10.1093/shm/hkx118PMC666459031384101

18.
For recent studies on the place of the material in the neurosciences, see the special issue of *Medical History*, 60, 3 (2016); and the contributions to *Science in Context*, 28, 1 (2015). For broader histories on the role of the material in the formation of scientific knowledge, see Annemarie Mol, *The Body Multiple: Ontology in Medical Practice* (Durham and London: Duke University Press, 2002); Hans-Jörg Rheinberger, *Toward a History of Epistemic Things: Synthesising Proteins in the Test Tube* (Stanford, CA: Stanford University Press, 1997); Henning Schmidgen, *The Helmholtz Curves: Tracing Lost Time*, trans. by Nils F. Schott (New York: Fordham University Press, 2014).

19.
See Wilfred Trotter, *Instincts of the Herd in Peace and War* (London: T.F. Unwin Ltd, 1919). For context, see Gillian Swanson, ‘Collectivity, Human Fulfilment and the “Force of Life”: Wilfred Trotter’s Concept of the Herd Instinct in Early 20th-Century Britain’, *History of the Human Sciences*, 27, 1 (2013), 21–50.

20.
See eg. Brain and Cairns, *op. cit.* (note 4), 668.

21.
Notwithstanding my reliance on his writings, Trotter’s influence probably also extended to correspondence and meetings with Symonds and other neurologists at the Oxford Military Hospital, as implied in The National Archives (TNA), FD 1/3243, ‘Head Injuries: Research by Dr W.R. Russell, Edinburgh’, Letter from Wilfred Trotter, 25 November 1931; TNA, FD 1/3243, ‘Head Injuries: Research by Dr W. R. Russell, Edinburgh’, Letter from unknown, 20 November 1931; Symonds, *Studies in Neurology*, *op. cit.* (note 1), 12.

22.
See Wilfred Trotter, ‘The scalp, skull and brain’, in C.C. Choyce (ed.), *A System of Surgery* (London, New York, Toronto and Melbourne: Cassell and Co. Ltd, 1914), 305–418.

23.
Wilfred Trotter, ‘The scalp, skull and brain’, in C.C. Choyce (ed.), *A System of Surgery*, 2nd edn (London, New York, Toronto and Melbourne: Cassell and Co. Ltd, 1923), 436–565; Wilfred Trotter and Julian Taylor, ‘The scalp, skull and brain’, in C.C. Choyce (ed.), *A System of Surgery*, 3rd edn (London: Waverly Book Company, 1932), 332–476.

24.

T.R. Elliott , ‘Wilfred Batten Lewis Trotter’, Obituary Notices of the Fellows of the Royal Society, 3, 9 (1941), 328–330.

25.

Wilfred Trotter , ‘An Address on the Management of Head Injuries’, The Lancet, 2, 5332 (1925), 955.

26.

Wilfred Trotter , ‘On Certain Minor Injuries of the Brain: Being the Annual Oration, Medical Society of London’, British Medical Journal, 1, 3306 (1924), 817.10.1136/bmj.1.3306.816PMC230436420771576

27.

*Ibid*., 816.

28.

Wilfred Trotter , ‘An Address on the Evolution of the Surgery of Head Injuries’, The Lancet, 1, 5552 (1930), 170.

29.
Trotter, *op. cit.* (note 26), 817.

30.

Geoffrey Jefferson , ‘The Nature of Concussion’, British Medical Journal, 1, 4330 (1944), 1–5.2078520110.1136/bmj.1.4330.1PMC2283190

31.

C.P. Symonds , ‘The Effects of Injury Upon the Brain’, The Lancet, 219, 5668 (1932), 821.

32.
Russell, ‘Cerebral Involvement in Head Injury’, *op. cit.* (note 7), 578 and *passim*.

33.
C.P. Symonds, ‘Observations on the Differential Diagnosis and Treatment of Cerebral States Consequent Upon Head Injuries’, *British Medical Journal*, 2, 3540 (1928), 829; no author, *op. cit.* (note 11), 295; Russell, ‘Cerebral Involvement in Head Injury’, *op. cit.* (note 7), 600.10.1136/bmj.2.3540.829PMC245640120774242

34.
For instance, see C.P. Symonds , ‘Prognosis in Closed Head Injuries’, British Medical Bulletin, 3, 1–3 (1945), 14.

35.
See Susan Lamb , ‘My Resisting Getting Well: Neurasthenia and Subconscious Conflict in Patient–Psychiatrist Interactions in Prewar America’, Journal of the History of the Behavioral Sciences, 52, 2 (2016), 124–145.2691615310.1002/jhbs.21777

36.
For instance, E. Farquhar Buzzard , ‘An Address on Traumatic Neurasthenia’, The Lancet, 2, 5233 (1923), 1285–1288.

37.
For example, C.P. Symonds, *op. cit.* (note 6), 71; William A. Brend, ‘Differential Diagnosis of Contusion of the Brain and Psychoneurosis’, *British Medical Journal*, 1, 4197 (1941), 885. I take the phrase ‘dumping ground’ from Ian Skottowe, ‘The Dumping Ground of Neurasthenia’, *The Lancet*, 215, 5550 (1930), 106.10.1136/bmj.1.4197.885PMC216208120783707

38.
See eg. Danson, *op. cit.* (note 1), 60.

39.
Russell was often heralded as the originator of this line of thinking, based on a set of follow-up studies he did at the Edinburgh Royal Infirmary in the early 1930s, a discussion of which was published as Russell, ‘The After-Effects of Head Injury’, *op. cit.* (note 7); Russell, *op. cit.* (note 1), esp. 585 and *passim* It should be noted, however, that Russell did not demur from the view that the desire for compensation could cause traumatic neurosis, merely that he found some follow-up cases that had long-term concussive sequelae despite no claim pending. See *ibid*, 134 and 142. Russell cautioned against making direct comparisons with his study, however; advice often ignored by his contemporaries, eg. Symonds, *op. cit.* (note 6), 102.

40.

Millais Culpin , ‘Some Cases of Traumatic Neurasthenia’, The Lancet, 2, 5631 (1931), 233–237.

41.

Edward Mapother , ‘Mental Symptoms Associated with Head Injury’, British Medical Journal, 2, 4012 (1937), 1060.10.1136/bmj.2.4012.1055PMC208776820781074

42.
See Danson, *op. cit.* (note 1), 60; the comments of Symonds in [no author], ‘Discussion on Differential Diagnosis and Treatment of Post-Contusional States’, *Proceedings of the Royal Society of Medicine*, 35, 9 (1942), 604 and 608.10.1177/003591574203500905PMC199830019992544

43.
For an overview of debates, see Trimble, *op. cit.* (note 16).

44.
See eg. Jefferson, *op. cit.* (note 3), 1 and *passim*.

45.
Symonds, *op. cit.* (note 6); no author, *op. cit.* (note 42), 601–7.

46.
For example, suggestion or unconscious motivations, of the sort identified by medico-legal examiners. For context, see Rhodri Hayward, *The Transformation of the Psyche in British Primary Care, 1880–1970* (London: Bloomsbury, 2014), ch. 2. On the growing role of neurologists in adjudicating on compensation claims post-1918, see Casper, *op. cit.* (note 13), 80–4.

47.
For positive references to Trotter’s work on concussion, see Symonds, *op. cit.* (note 6), 71; Symonds, *op. cit.* (note 34), 14; the comments in no author, *op. cit.* (note 11), 295.

48.
Symonds, *op. cit.* (note 6), 97.

49.
See his comments in no author, *op. cit.* (note 11), 299.

50.
As Symonds wrote: ‘Our problem begins with the reconstruction of the story of the injury, often with scant information apart from what the patient can tell us.’ See his comments in no author, *op. cit.* (note 42), 601. For a similar argument, see Geoffrey Jefferson, ‘The Acute Head Injury as a Neurological Problem’, *Glasgow Medical Journal*, 20, 3 (1942), 78.

51.
See the comments of Symonds in no author, *op. cit.* (note 42), 601; the implied criticism of surgeons in Russell, ‘Cerebral Involvement in Head Injury’, *op. cit.* (note 7), 600; Symonds, *op. cit.* (note 5), 320–1. Russell further complained that sometimes, if the patient presented with multiple injuries, only the most severe was written on the patient-notes; often, in other words, neurologists had to infer whether a patient had suffered from concussion based on a reading of their neurological symptoms. See Russell, ‘Cerebral Involvement in Head Injury’, *op. cit.* (note 7), 555.

52.
Symonds, *op. cit.* (note 34), 15. While early psychometric tests were seen as useful to used later to compare a patient’s progress, Russell observed that they were not always needed, that simple tests and talking with relatives was usually more insightful. See W. Ritchie Russell, ‘Studies on Head Injury’, *British Medical Bulletin*, 10, 1 (1954), 66. Symonds also found talking to relatives fruitful. See Symonds, *Studies in Neurology*, *op. cit.* (note 1), 124.

53.
Symonds, *op. cit.* (note 5), 321. In 1928, Symonds had also written of the need for proper documentation of predisposing issues with a patient, which, he claimed, surgeons and physicians never bothered to record. See Symonds, *op. cit.* (note 33), 831.

54.
Jefferson, *op. cit.* (note 50), 78. Symonds further proposed that neurologists were best placed to acquire this information and add it to the patient’s chart. See Symonds, *op. cit.* (note 5), 326.

55.
From the literature review offered by Russell, it would appear that neurologists had little interest in concussion prior to the 1930s, with the twentieth-century literature dominated by physiologists and general surgeons (eg. Trotter). See Russell, ‘Cerebral Involvement in Head Injury’, *op. cit.* (note 7), 550. A similar point is also made in Cairns, *op. cit.* (note 12), 869. Also note the comments of Jefferson as to the lack of regard in which neurosurgery was held prior to the War, as quoted in Tailor and Handa, *op. cit.* (note 9), 190.

56.
On tensions between neurologists and neurosurgeons, see Casper, *op. cit.* (note 13), esp. chs 3 and 4.

57.
On the dichotomy between <24 hours and >24 hours patients, and its impact on professional roles, see the distinction imposed in Symonds, *op. cit.* (note 33), 829–31. On concussion as a neurological (and not surgical) issue, see Jefferson, *op. cit.* (note 50), 77; Symonds, *op. cit.* (note 33), 829.

58.
On the value of neurological observation of the concussed, see Symonds, *op. cit.* (note 5), 321.

59.
On the importance of documentation, see the comments on clinical records in TNA, FD 1/5342, ‘Memorandum on the Treatment of Head Injuries in War’, 2. This memo was unauthored, but written by Cairns as the minutes for the Brain Injuries Committee record that he introduced the discussion of it (see TNA, FD 1/5342. ‘Brain Injuries Committee’, 7 March 1940, 2).

60.
Although Russell was often identified as the first to use PTA to in the assessment of head trauma, it is clear that he was heavily influenced by Symonds. See his comments in Russell, ‘Cerebral Involvement in Head Injury’, *op. cit.* (note 7), 552.

61.
Russell, ‘Cerebral Involvement in Head Injury’, *op. cit* (note 7), 533; Russell’s comments in [no author], ‘Discussion on the Diagnosis and Treatment of Acute Head Injuries’, *Proceedings of the Royal Society of Medicine*, 25, 5 (1932), 751–52.

62.
Symonds, *op. cit.* (note 6), 76. This marked a development from previous comments made by Russell, who merely stated that PTA was useful in that it could be used long after an accident if needed. See Russell, ‘Cerebral Involvement in Head Injury’, *op. cit.* (note 7), 554.

63.
Russell, ‘The After-Effects of Head Injury’, *op. cit.* (note 7), 135.

64.
Cross-examination was the best way to determine the stage that the patient was at, according to Russell. See Russell, ‘Cerebral Involvement in Head Injury’, *op. cit.* (note 7), 553.

65.

*Ibid*, 552 and 596; C.P. Symonds and W. Ritchie Russell , ‘Accidental Head Injuries: Prognosis in Service Patients’, The Lancet, 241, 6227 (1943), 7.

66.
Cf. Casper on the neurological belief that, with recourse to physiological and anatomical tests, the neurological exam ‘turned the patient’s body into a machine of its own reading’ in Casper, *op. cit.* (note 11).

67.
See G.F. Rowbotham, ‘The Long-Term Results of Injuries of the Head (A Medical, Economical and Sociological Survey)’, *British Journal of Psychiatry*, 95, 399 (1949), 339 and *passim* Indeed, many neurologists expressed disappointment that no one objective method could diagnose concussive sequelae, eg. see the comments in Symonds, *op. cit.* (note 5), 326.

68.
Symonds, *op. cit.* (note 6), 101.

69.
For neurologists’ frustrations with terminology, see no author, *op. cit.* (note 42), 601; Symonds, *op. cit.* (note 5), 321; and Symonds’s complaint regarding the lack of objective standards for measuring amnesia, and the effect of this on neurologists, in Symonds, *op. cit.* (note 1), 119. Or, as Russell wrote, the two features of concussion (a return to normalcy and no evidence of visible pathology) could rarely be both proven in the one case with ease. Russell, *op. cit.* (note 52), 65.

70.
On which, see Symonds, *op. cit.* (note 6), 76; Jefferson, *op. cit.* (note 3), 3; Mapother, *op. cit.* (note 41), 1060.

71.
Symonds, *op. cit.* (note 5), 321. The minutes for the Brain Injuries Committee would appear to support Symonds’s claim. In May 1940, the Committee recommended that a set of “‘psychological” terms’ be produced in order to ‘define in a succinct manner certain states of functional disorder’. See TNA, FD 1/5342, ‘Brain Injuries Committee’, 2nd May 1940, 3. For the final list of terms, see Medical Research Council Brain Injuries Committee, *A Glossary of Psychological Terms Commonly Used in Cases of Head Injury* (London: HMSO, 1941).

72.
Both Cairns and Symonds had petitioned for its creation as early as 1938. See the correspondence in TNA, AIR 2/3948, ‘Treatment of Head Injuries at Military Hospital, St Hugh’s College, Oxford in Time of War, 1938–43’. On Cairns’s role in petitioning for the creation of a neurology department at Oxford, see Fraenkel, *op. cit.* (note 9), chs 8 and 9. Note that Russell had no hand in the planning of the hospital, but was recruited from the Edinburgh Royal Infirmary by the time of the former’s opening in 1940.

73.
The Military Hospital originally housed 100 beds, though this number rose to 300 after one year once additional buildings were erected on the college grounds. The growth of the number of beds in the hospital occurred around 1941. From August 1941 onwards, the process of indexing and cataloguing patient-records (discussed below) ceased as it was no longer ‘practicable’ to study every patient, only the interesting ones. See TNA, FD 1/5342, ‘Report from Oxford Military Hospital (Head Injuries)’, undated, 2; TNA, WO 222/845, Derek Denny-Brown, ‘Special Report Submitted in Conformity with Southern Command Medical Routine Orders, o1/1940’, 22 October 1940, para 410.

74.
For instance, Cairns had a long-standing interest in the accumulation of neurological patient-records for research purposes, as is evident in his attempts to found and develop the medical school at Oxford in the 1930s. See Fraenkel, *op. cit.* (note 9), 125.

75.
The first meeting of the Committee included Norman Dott, J.G. Greenfield, Geoffrey Jefferson, Aubrey Lewis, George Riddoch and E.A Carmichael, in addition to Cairns and Symonds. The Committee was chaired by E.D. Adrian. See TNA, FD 1/5342. ‘Brain Injuries Committee’, 7 March 1940.

76.
TNA, FD 1/5342, ‘Memorandum on Plans for Research at the Oxford Military Hospital (Head Injuries) and Application for Grant by Group-Capt. Symonds’, undated, 1. For insight into the specific areas that Committee members suggested, see TNA, FD 1/5342, ‘Suggested Problems for Discussion’, undated.

77.
‘Memorandum on Plans for Research at the Oxford Military Hospital (Head Injuries) and Application for Grant by Group-Capt. Symonds’, *op. cit.* (note 76), 1. Symonds began advocating for additional resources for administrative support at the Military Hospital from December 1939 onwards. However, his early overtures to the Army, who ran the Military Hospital, and to the MRC were rebuffed. The MRC would only entertain Symonds’s request if it were part of a broader attempt to help coordinate neurological research. The Brain Injuries Committee of the MRC was thus born. On Symonds’s early rejections, and for his initial work in identifying Committee members, see TNA, FD 1/6138, ‘Brain Injuries Committee: Constitution and Membership, 1939–1943’; and some of the correspondence contained in TNA, FD 1/6136, ‘Wounds and the Nervous System: Research at Oxford, 1939–41’.

78.
Symonds’s grant application was initially reviewed by the Brain Injuries Committee, who recommended its funding to the MRC as noted in ‘Brain Injuries Committee’, *op. cit.* (note 75), 4.

79.
See St Hugh’s College Archives, HHA/4/2/2, ‘Letter from Hugh Cairns to Geoffrey Jefferson’, 17 July 1940.

80.
The minutes for the Brain Injuries Committee in this period suggest that neurologists were concerned that military records would be reclaimed by the Army or pension authorities at a later date, hence why the patient-records at the Military Hospital were to be compiled and stored separately. See Symonds in TNA, FD 1/6137, ‘Letter from C.P. Symonds to F.J.C. Herald’, 29 February 1944; and the comments of Geoffrey Jefferson in ‘Brain Injuries Committee’, *op. cit.* (note 75), 2.

81.
This list is not exhaustive.

82.
The list of medical criteria for collection is contained in TNA, FD 1/5341, ‘Case-Taking Scheme in Use at the Oxford Military Hospital (Head Injuries)’, undated. Further, more detailed particulars of what Symonds wanted medics to record can be found in an unnamed, undated and unauthored report in TNA, WO 222/845. The topicality of this report would suggest that it is was written by Symonds and distributed to the medical officers at Oxford at some stage upon the hospital’s opening.

83.
For the information that Symonds wanted physicians to record on patients, and the means of how they were to acquire it, see TNA, FD 1/5342, ‘Report from the Oxford Military Hospital (Head Injuries)’ and ‘Guide to Index’, undated. This report was not authored, but I know that it was written by Symonds (as, in it, he refers to himself as the ‘grant-holder’ of the MRC funding). Symonds also regularly reviewed charts to ensure uniformity.

84.
For an exact breakdown of the patient-data that was indexed, see TNA, FD 1/5342, ‘Routine Scheme for Examination and Case Records’, undated. As Symonds later explained: ‘The object [of research at Oxford] is to accumulate a well-documented and systematically indexed collection of clinical data from the material afforded by this hospital with its hitherto unique opportunities.’ TNA, FD 1/6137, ‘Letter from C.P. Symonds to the Secretary of the Medical Research Council’, 15 October 1943.

85.
One of the primary foci of Symonds’s research in this period was the relationship between predisposition and the severity of neurotic sequelae. For his comments on the subject, see Symonds, *op. cit.* (note 5), 326; Symonds, *op. cit.* (note 6), 100; and Symonds and Russell, *op. cit.* (note 65), 9. On the influence of Symonds on the collection of patient-data at Oxford with a view to assessing predisposition, see the comments of Russell in TNA, FD 1/5343, Dr W. Ritchie Russell, ‘Analysis of the First Thousand Cases of Head Injury Admitted to the Military Hospital for Head Injuries’, 2. Symonds had aspirations for the Oxford data-set, hoping that the patient-records, when studied, revealed a positive relationship between predisposition and prognosis. See ‘Report from the Oxford Military Hospital (Head Injuries)’, *op. cit.* (note 73), 1.

86.
On the use of paper-technologies to standardise neurological research, and thereby obscure local idiosyncrasies and replace them with ‘universal’ standards, see Susan Leigh Star, ‘Scientific Work and Uncertainty’, *Social Studies of Science*, 15, 3 (1985), 399 and *passim*.10.1177/03063128501500300111620769

87.
As medical officers compiling patient-records were warned: ‘Subjective and objective data should not be mixed up either.’ See the untitled, unauthored and undated document contained in TNA, WO 222/845. The topicality of this document would suggest that it was written by Symonds.

88.
I should issue a note of caution when describing the organisation of patient-data at Oxford as a ‘database’: databases typically organise pre-existing data that is independent from the database. As the discussion below makes clear, the medics at Oxford sought to collect patient-data in expectation that it would eventually be turned into a database (ie. by being indexed onto punch-cards).

89.
See Russell, ‘Analysis of First Thousand Cases of Head Injury Admitted to the Military Hospital for Head Injuries’, *op. cit.* (note 85), 1. Note Russell’s reference to ‘uniform assessment[s]’, and relate this back to my discussion of standardisation, above.

90.
Technically, it was the first 1103 patients admitted to the hospital. In their publications, however, neurologists simply referred to this group as the ‘first 1000’. They also indexed another 300 or so patients ‘of interest’ in the same way as the first 1000, but retained their records in a separate series. See TNA, FD 1/6137, C.P. Symonds, ‘Progress Report, 1941–42’, 5 September 1942.

91.
This is not an exhaustive list.

92.
The index-cards are contained in St Hugh’s College Archives, University of Oxford, HHA/2/1, ‘Military Hospital Punch Cards, c. 1940–48’. I assume that the cards were stored in a drawer, filing-cabinet or box, but I have not encountered a specific description in the primary sources.

93.
Alternatively, a researcher may identify a patient by their military-number or by the ‘MRC Number’ that the Oxford researchers gave to the first 1000 patients involved in the MRC-funded project to better organise their records. The use of these numbers meant that the punch-cards could be reassembled or removed from storage for the purposes of identifying a patient-record, and then returned later and in the correct order. On the flexibility of index-cards as a research-technology, see Isabella Charmantier and Staffan Müller-Wille, ‘Carl Linnaeus’s Botanical Paper Slips (1767–73)’, *Intellectual History Review*, 24, 2 (2014), 217.

94.
‘Report from Oxford Military Hospital (Head Injuries)’, *op. cit.* (note 73), 1.

95.
I refer here of the work of Lishman and Newcombe. On the former’s research on gun-shot wounds to the head, see his reminiscences in Michael Salzberg, ‘A Pioneer of Neuropsychiatry: Professor Alwyn Lishman’, *Australian and New Zealand Journal of Psychiatry*, 38, 1–2 (2004), 2–9. For Newcombe’s work on neurolinguistics, see Freda Newcombe, ‘The Psychological Consequences of Closed Head Injury: Assessment and Rehabilitation’, *Injury*, 14, 2 (1982), 111–36.

96.
See eg. Symonds’s comments in no author, *op. cit.* (note 42), 605–6.

97.
The correspondence for which can be found in St Hugh’s College Archives, HHA/4/2/5.

98.

Hugh Cairns , ‘Planning for Treatment of Head Injuries’, British Medical Journal, 1, 4288 (1943), 313.2078472710.1136/bmj.1.4288.313PMC2282444

99.
Symonds and Russell, *op. cit.* (note 65), 7.

100.
Russell, *op. cit.* (note 1), 521. This article was originally delivered as a lecture.

101.
This was Symonds and Russell, *op. cit.* (note 65). An earlier pair of papers, both written by Russell and with PTA at their centre, was circulated to the Brain Injuries Committee. I suspect that these were later rewritten with Symonds. For the papers, see TNA, FD 1/5343, ‘Report from Ritchie Russell, Military Hospital (Head Injuries)’, St.Hugh’s College, Oxford, 10 August 1942; TNA, FD 1/5343, ‘Analysis of First Thousand Cases of Head Injury Admitted to the Military Hospital for Head Injuries (Explanatory Text to Paper B.I.C. 61 by Dr W. Ritchie Russell)’. For the positive reception of these papers to the Brain Injuries Committee, see the brief comments in TNA, FD 1/5269, ‘Brain Injuries Committee’, 12 March 1942, 2.

102.
For more general comments on how databases are founded, and what problems this can create when the object of study does not yield to the planned system of classification, see Christine Hine, ‘Databases as Scientific Instruments and Their Role in the Ordering of Scientific Work’, *Social Studies of Science*, 36, 2 (2006), 278 and *passim*.

103.
See Trimble, *op. cit.* (note 5).

104.
On the postwar period, see Ross, *op. cit.* (note 25).

